# Resting state brain subnetwork relates to prosociality and compassion in adolescents

**DOI:** 10.3389/fpsyg.2022.1012745

**Published:** 2022-10-20

**Authors:** Benjamin S. Sipes, Angela Jakary, Yi Li, Jeffrey E. Max, Tony T. Yang, Olga Tymofiyeva

**Affiliations:** ^1^Department of Radiology and Biomedical Imaging, University of California, San Francisco, San Francisco, CA, United States; ^2^Department of Psychiatry, University of California, San Diego, San Diego, CA, United States; ^3^Rady Children’s Hospital San Diego, San Diego, CA, United States; ^4^Department of Psychiatry and Behavioral Sciences, The Langley Porter Psychiatric Institute, Division of Child and Adolescent Psychiatry, Weill Institute for Neurosciences, University of California, San Francisco, San Francisco, CA, United States

**Keywords:** MRI, connectivity (graph theory), adolescence, prosocial, social neuroscience, compassion, resting state, network based statistics

## Abstract

Adolescence is a crucial time for social development, especially for helping (prosocial) and compassionate behaviors; yet brain networks involved in adolescent prosociality and compassion currently remain underexplored. Here, we sought to evaluate a recently proposed domain-general developmental (Do-GooD) network model of prosocial cognition by relating adolescent functional and structural brain networks with prosocial and compassionate disposition. We acquired resting state fMRI and diffusion MRI from 95 adolescents (ages 14–19  years; 46 males; 49 females) along with self-report questionnaires assessing prosociality and compassion. We then applied the Network-Based Statistic (NBS) to inductively investigate whether there is a significant subnetwork related to prosociality and compassion while controlling for age and sex. Based on the Do-GooD model, we expected that this subnetwork would involve connectivity to the ventromedial prefrontal cortex (VMPFC) from three domain-general networks, the default mode network (DMN), the salience network, and the control network, as well as from the DMN to the mirror neuron systems. NBS revealed a significant functional (but not structural) subnetwork related to prosociality and compassion connecting 31 regions (*p* = 0.02), showing DMN and DLPFC connectivity to the VMPFC; DMN connectivity to mirror neuron systems; and connectivity between the DMN and cerebellum. These findings largely support and extend the Do-GooD model of prosocial cognition in adolescents by further illuminating network-based relationships that have the potential to advance our understanding of brain mechanisms of prosociality.

## Introduction

Prosocial behavior, defined as compassionate actions that benefit others, is associated with improved physical health in the giver (Inagaki and Eisenberger, 2016; Moieni et al., 2019), and while childhood prosocial behavior predicts adulthood prosociality (Eisenberg et al., 2001), the brain mechanisms of prosociality and compassion during neurodevelopment remain poorly understood. Better understanding these mechanisms may help improve educational and mental health interventions that promote prosocial and compassionate behavior in youth.

Functional magnetic resonance imaging (fMRI) studies investigating adolescent prosocial behavior have mostly focused on identifying brain regions with significant activation during prosocial tasks. This research generally operationalized prosociality through the amount of resources (e.g., money) given to another person versus themselves (van den Bos et al., 2009, 2011; Telzer et al., 2011, 2013; Gunther Moor et al., 2012; Güroglu et al., 2014; Van Hoorn et al., 2016; Will et al., 2016, 2018; Sakai et al., 2017; Schreuders et al., 2018, 2019; Tousignant et al., 2018; Spaans et al., 2020; Brandner et al., 2021; Duell et al., 2021). Two studies analyzed neural correlates of trait-based prosociality related to prosocial questionnaires, namely the Strengths and Difficulties Questionnaire (SDQ) prosocial sub-scale (Goodman, 2001), indicating a relationship to seed-based resting state connectivity (Okada et al., 2019) and to longitudinal changes in cortical thickness (Ferschmann et al., 2019). Together, these findings implicate a broad set of brain regions largely grouped by three networks: default mode network (DMN) regions—the medial prefrontal cortex (MPFC), the posterior cingulate cortex (PCC), precuneus, and temporal–parietal junction (TPJ); salience network (SN) regions—the anterior cingulate cortex (ACC) and insula; and control network (CN) regions—the dorsolateral prefrontal cortex (DLPFC) and the inferior parietal lobule (IPL; For a recent review, see Sipes et al., 2022). However, regions deemed significant for prosociality varied between task paradigms, highlighting the need for additional research to clarify the role of regions and networks in adolescent prosociality.

Recently, we proposed a domain-general developmental “Do-GooD” network model of prosocial cognition in adolescents (Sipes et al., 2022) based on works referenced above. According to the Do-GooD model, three domain-general networks—the default mode network (DMN), the control network (CN), and the salience network (SN)—contribute value computations for prosocial decisions, which are integrated in the ventromedial prefrontal cortex (VMPFC). We proposed that the DMN, supported by mirror neuron systems, computes value for self and other and sends it to the VMPFC. We suggested that these computations can be influenced by events that occur before prosocial decision-making and may reflect general prosocial disposition. Similarly, we proposed that the CN develops during adolescence and contributes value for social norms and moral attitudes which are sent to the VMPFC. Specific predictions of the Do-GooD model are that prosocial disposition (i.e., outside of tasks) should positively relate to connectivity within the DMN (reflecting a developed self versus other value-computation system) and connectivity to the VMPFC from the DMN and CN (reflecting that value’s accrual). In the Do-GooD model, the SN accounted for self-monitoring ongoing fairness across task sessions that, in classic block-based fMRI design, required many repetitions of prosocial decision-making. The SN’s involvement in self-monitoring *during tasks* suggests that its core regions, such as the anterior cingulate cortex and insula, may *not* be related to prosociality *outside of tasks* (Sipes et al., 2022).

Here, we sought to evaluate these predictions inductively. Since the Do-GooD model was mostly inferred from task-based activation fMRI studies and not connectivity studies, we believed an important first step in exploring how brain networks relate to adolescent prosociality was to use a data-driven whole-brain technique that would derive significant relationships without limiting the analysis to only a few connections. The Network-Based Statistic (NBS) is such a technique with the power to inductively interrogate the relationship between brain *networks* and psychometric data while non-parametrically correcting for multiple comparisons (Zalesky et al., 2010). NBS is designed to leverage the full high-dimensionality of brain networks to derive subnetworks related to (in this case) adolescent prosociality and compassion that are unlikely to arise in randomized networks with similar global properties.

Using NBS, we tested whether resting state functional and structural brain networks contain subnetworks related to prosocial behavior and compassion during adolescence. We expected that, if such subnetworks existed, they would reveal relationships hypothesized by the Do-GooD model of prosocial cognition; namely that there should be increased within-DMN connectivity, greater connectivity between the VMPFC and the DMN and CN, and that these relationships would be reflected by subnetwork connections with key DMN and CN regions previously related to task-based prosocial behavior, including the medial prefrontal cortex, the precuneus, the dorsolateral prefrontal cortex, and the temporal parietal junction.

## Materials and methods

### Participants

Ninety-five healthy adolescents (mean 16.0 ± 1.3 years, range: 14–19 years., 49 females, 46 males) were recruited from a community sample in Northern California. Adolescent participants were recruited using University of California, San Francisco (UCSF) IRB-approved flyers posted around the UCSF campus, neighboring areas, and local high schools. Recruitment also involved online posts to parent communities and word-of-mouth referrals to the study. Adolescents were excluded from the study if they reported MRI contraindications, psychiatric diagnoses, or were taking any psychotropic medications. This study was approved by the UCSF Institutional Review Board (IRB). Each participant completed two self-report questionnaires and underwent a 3T MRI scan.

### Study questionnaires

All participants completed the Strengths and Difficulties Questionnaire (SDQ) and the Compassionate Engagement and Action Scales for Youth (CEASY).

The SDQ measures prosociality through a *prosocial behavior* sub-scale that assesses adolescents’ general patterns of prosocial behavior (Goodman, 2001). This three-point Likert scale gives examples of prosocial actions and asks adolescents to rate the extent to which each statement is true (not true = 0, somewhat true = 1, or certainly true = 2). The total score is the based on the responses to five questions, with total scores ranging 0–10.

The CEASY has adolescent validated subscales for compassionate engagement and action for self, for others, and as perceived from others (Gilbert et al., 2017). We used the total sub-score regarding *compassion for others* as a measure of outwardly compassionate tendencies. The subscale’s ten items were scored on a Likert scale from 1 to 10, with the total scores ranging 10–100.

Importantly, the SDQ measures real-life examples of prosocial action while the CEASY measures compassionate cognitive engagement and generalized actions toward others. Since these questionnaires measure different aspects of a similar psychological construct (prosocial actions and compassionate engagement), we sought to identify a subnetwork involving the interaction (product) of these scores. This projects both scores into a common space, and it allows for each super-score to be a more granular measure of this general positive-psychological phenomenon. Since no participant scored 0 on the SDQ prosociality sub-scale, no super-scores were collapsed to 0.

### Magnetic resonance imaging acquisition

All participants underwent 3T (GE) MRI scans, which included a standard T1-weighted sequence (MPRAGE sequence, with TR/TI/TE = 10.2 s/450 ms/4.2 s, flip angle = 15°, 1 mm isotropic resolution, ASSET acceleration factor = 2, acquisition time = 6 min and 11 s), a T2*-weighted resting state fMRI sequence (TR = 800 ms, TE = 30 ms, FOV = 21.6 cm, 90×90 matrix, 2.4 mm isotropic resolution, 525 time-points, acquisition time = 7 min), and a 30-direction diffusion tensor imaging (DTI) sequence (TR = 7,500 ms, TE = 60.7 ms, FOV = 25.6 cm, 128 × 128 matrix, and slice thickness = 2 mm resulting in a 2 mm isotropic resolution, *b* = 1,000s/mm2, acquisition time = 4 min).

### Functional connectivity

Functional connectomes were constructed using the default processing pipelines from the functional connectivity toolbox (CONN; Whitfield-Gabrieli and Nieto-Castanon, 2012) and SPM12 (Wellcome Department of Imaging Neuroscience, London, United Kingdom).^1^ Briefly, T2*-weighted images were aligned to a common space and underwent motion estimation and correction as well as slice-timing correction. Outlier scans were identified for scrubbing, and gray matter in the T1-weighted images and T2*-weighted images were segmented and normalized. The functional data was smoothed using an 8 mm FWHM kernel. Confounds were regressed out of the data, including white matter signal, cerebrospinal fluid (CSF) signal, realignment, scrubbing, and the effect of rest. The brain was parceled into 116 regions of interest (ROIs) by the Automated Anatomical Labeling (AAL) atlas (Tzourio-Mazoyer et al., 2002). ROI-to-ROI functional connectivity (FC) was computed as the Fisher’s Z transformed Pearson’s correlation of mean BOLD time series within each region. All FC networks were thresholded to retain only positive correlations as network weights.

### Structural connectivity

Structural connectomes were constructed as described previously (Yuan et al., 2019). Briefly, we segmented the T1-weighted image cortex, subcortex, and cerebellum into a total of 116 ROIs using the AAL atlas, then registered it to DTI space. We used deterministic tractography with the Diffusion Toolkit (Wang et al., 2007) to generate tractograms for each participant. The network was constructed by considering each AAL ROI as a network node, and connections between nodes were weighted by the average fractional anisotropy along all reconstructed streamlines between them.

### Network-based statistic analysis

The Network-Based Statistic (NBS) toolbox was used to identify significant functional and structural subnetworks related to prosociality and compassion while correcting for multiple comparisons (Zalesky et al., 2010). Our design matrix was structured as [constant = 1; SDQ*CEASY; Age; Sex], and our contrast vector was [0 1 0 0] to assess the effect of prosociality and compassion while controlling for age and sex. Our inputs into NBS were the 116 × 116 functional connectivity and structural connectivity matrices, stacked across all 95 participants. Because NBS uses a test-statistic to determine significant subnetwork edges, we first swept through a range of test-statistic thresholds on the interval between 2 and 3 in 0.05 steps to determine the test-statistic that minimized the subnetwork size while maintaining significance for multiple comparisons compared to 2,000 randomized-network permutations. Based on these results, we ultimately used a *t*-test comparison with a test-statistic = 2.75 and with 10,000 randomized-network permutations to obtain the final value of p for the subnetwork related to prosociality and compassion. Post-hoc analysis sought to verify this relationship by testing the correlation between each subject’s subnetwork weight (sum of all subnetwork edges) and their combined prosociality and compassion score. For interpretability, we quantified the subnetwork’s modular clustering of regions through the Louvain community detection algorithm (*γ* = 0.8) implemented in the Brain Connectivity Toolbox (Rubinov and Sporns, 2010).

## Results

### Questionnaire results

Adolescents in our study exhibited a range of prosocial tendencies, but our sample leaned toward being more prosocial. The means (standard deviations) for the questionnaires are as follows: SDQ-Prosocial = 8.23 (1.48); CEASY-For Others = 76.86 (14.56); SDQ-Prosocial*CEASY-For Others = 644.45 (190.45).

### Functional connectivity results

**Figure 1:**
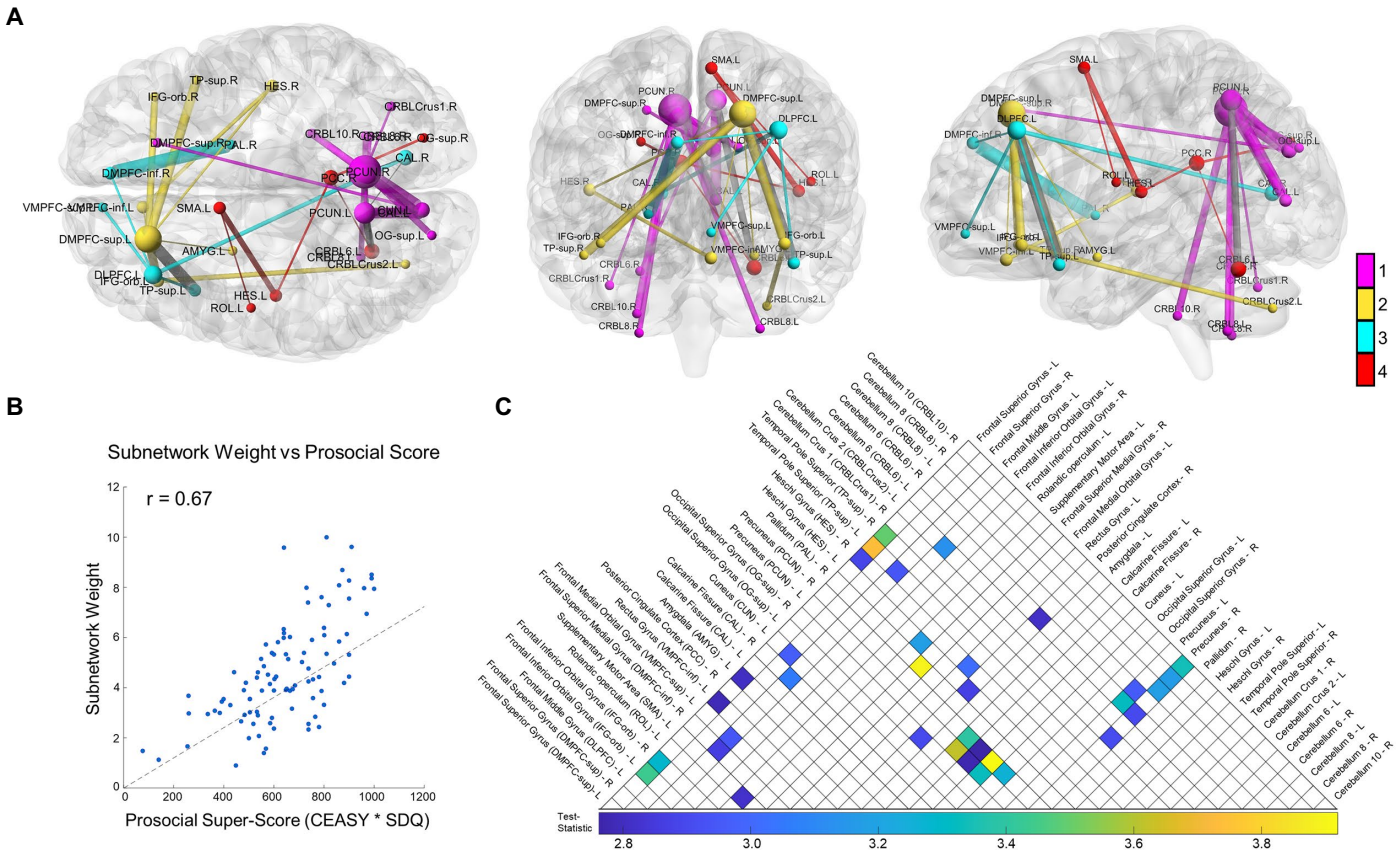
Study results. We show the overall results from the Network-Based Statistic (NBS) with resting state functional connectivity related to prosociality and compassion. **(A)** The NBS identified subnetwork is depicted from three orthogonal directions, from left to right: axial, coronal, and sagittal. For visualization, node size scales relative to that node’s degree (number of connections) and edge thickness scales relative to that edge’s test-statistic (relationship strength to prosociality and compassion). Network modules were identified using the Louvain community detection algorithm, and the nodes and edges are colored relative to their module (inter-modular edges shown in gray). In summary, the module shown in magenta centers on the bilateral precuneus and connects with the right DMPFC-inf, bilateral occipital lobe and cerebellum; the yellow module centers on the left DMPFC-sup and connects to regions including the bilateral IFG, left amygdala, and bilateral TP-sup, right Heschl’s gyrus, and left VMPFC; the cyan module centers on the left DLPFC and connects to regions including the right DMPFC-inf, left TP-sup, right calcarine fissure, and right pallidum; finally the red module is more central, connecting the PCC, to the occipital lobe, left Heschl’s gyrus, the Rolandic operculum, and the SMA. **(B)** A scatter plot showing the relationship between the total subnetwork weight (sum of all functional connectivity edge weights in the subnetwork) and the prosocial and compassion questionnaire scores. The dotted line shows the least-squares regression fit. The Pearson’s correlation is highly significant (*r* = 0.67, *p* < 0.0001). **(C)** An upper-triangle of the adjacency matrix depicting the same network shown in **(A)**, with a list of all subnetwork regions defined by the AAL atlas along their respective row/column indices. The matrix entries visualize the edge test-statistic with colored values based on the underlying color bar. Brain networks in this figure were visualized with BrainNet Viewer (Xia et al., 2013).

NBS revealed a statistically significant connected functional subnetwork positively associated with prosocial behavior and compassion in adolescents (*p* = 0.021; Figure 1A). For visualization purposes, we colored the subnetwork based on its module assignment using the Louvain community detection algorithm, scaled the size of each node relative to its degree, and scaled the thickness of each edge relative to its test-statistic. Post-hoc analysis on the subnetwork’s weight (sum of all functional connectivity edge weights in the subnetwork) confirmed a significant positive correlation with prosocial tendencies (*r* = 0.67, *p* < 0.0001; Figure 1B).

The subnetwork brain regions consisted of the following: the prefrontal lobe, including the left ventromedial prefrontal cortex (VMPFC), bilateral dorsomedial prefrontal cortex (DMPFC), left dorsolateral prefrontal cortex (DLPFC), bilateral orbital inferior frontal gyrus (IFG-orb), the Rolandic operculum, and the supplementary motor area (SMA); the temporal lobe including the bilateral superior temporal poles (TP-sup), bilateral Heschl’s gyrus (auditory cortex), and the left amygdala; medial parietal regions including the right posterior cingulate cortex (PCC) and bilateral precuneus; the occipital lobe including the bilateral superior occipital gyri (OG-sup) and calcarine fissures; one basal ganglia region, the right pallidum; many subregions of the cerebellum including Crus1 (right), Crus2 (left), regions VI (bilateral), VIII (bilateral), and X (right). The full list of AAL regions comprising the subnetwork is listed in Figure 1C, alongside their respective rows/columns in the adjacency matrix.

The Louvain community detection algorithm revealed that the subnetwork separated into four modules: a posterior community with many connections between the bilateral precuneus, occipital cortex, and the cerebellum; two anterior communities centered on the left DLPFC and left DMPFC-sup and connecting the prefrontal and temporal lobe regions, and a central community connecting the right PCC with the right OG-sup and left cerebellum VI with Heschl’s gyrus, Rolandic operculum, and the SMA. Interestingly, these results showed an overall left-leaning lateralization in the frontal cortex and some suggestion of regional homologues with module specificity, such as the right Heschl’s gyrus being involved in the left DMPFC-sup module and the left Heschl’s gyrus contributing to the SMA and PCC module.

**Figure 2:**
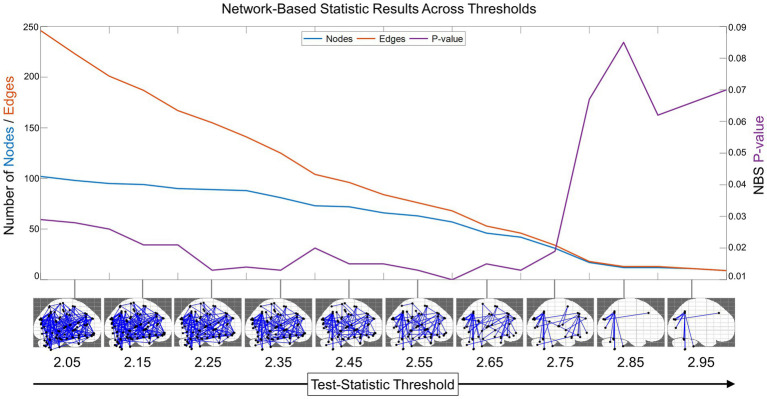
Network-based statistic (NBS) optimal threshold and sensitivity. To identify the optimal test-statistic threshold for the minimal connected subnetwork that was still significant after multiple comparisons, we computed the NBS results (with 2,000 randomized network permutations) across the interval from 2 to 3 with step size = 0.05 (for visualization purposes, NBS results in the sagittal plane are shown from 2.05 to 2.95). This revealed that the functional connectivity network has a subnetwork related to prosocial and compassionate trait-based measures that was enduring and significant across many test-statistic thresholds. The decreasing number of edges is shown in orange, the number of nodes in blue (scale on left y-axis), and the change in NBS-corrected value of p is shown in purple (scale on right y-axis). This plot reveals the optimal minimal subnetwork threshold to be test-statistic = 2.75.

We also found that NBS was sensitive to identifying a functional connectivity subnetwork across many test-statistic thresholds on the interval 2–3 (Figure 2). At the test-statistic threshold = 2, the subnetwork identified was significant and extensive, containing 102 out of 116 total AAL region nodes and 246 edges connecting them. As the threshold increased, edges that were weakly related to prosociality and compassion were pruned, retaining the largest connected significant subnetwork. Compellingly, all these networks were statistically significant (*p* < 0.05) on the interval from 2 to 2.75. Thus, a test-statistic of 2.75 was empirically revealed as the optimal test-statistic threshold for minimizing the resting state subnetwork size while maintaining a multiple-comparisons corrected significant value of *p* (Figure 2). Test-statistic thresholds greater than 2.75 disconnected the subnetwork and were no longer significant.

For completeness, we tested the negative association contrast and found no significant subnetworks (*p* = 0.54). We additionally tested whether significant subnetworks existed for each questionnaire, the SDQ and CEASY, individually. These results were non-significant with the CEASY showing one subnetwork (*p* = 0.28) and the SDQ showing two (*p* = 0.29 and *p* = 0.065; Supplementary Figure 1).

### Structural connectivity results

NBS revealed no significant structural connectivity subnetwork(s) related to prosociality and compassion in adolescents.

## Discussion

In the present study, we used an inductive data-driven whole-brain approach to investigate the brain network relationships to trait-level prosociality and compassion in adolescents (14–19 years). We found a resting state subnetwork related to prosociality and compassion in adolescents, enduring across many thresholds, controlling for differences in sex and age, and correcting for multiple comparisons using NBS. To our knowledge, our work is the first report of a resting state functional subnetwork related to adolescent prosociality.

Without *a priori* assumptions, adolescent resting state brain networks revealed a subnetwork related to prosociality that shows increased within-DMN connectivity, VMPFC connectivity with the DMN and CN, and connections between the DMN and CN. The DMN also shows significant connectivity to sensory/mirror neuron systems and to the cerebellum, suggesting that the DMN interfaces between these additional functional modules to support prosocial tendencies. It is possible these heterogeneous network relationships may in part be due to the multiple constructs involved in prosociality. Dunfield’s (2014) formulation suggests that there are three components facilitating prosocial behavior: perspective taking, problem recognition, and motivation to help. We may speculate that different modules of the identified resting state subnetwork relate to these components. For example, it could be that the DMN-sensory module is related to perspective taking while the left DLPFC module contains social-norm information that allows for problem recognition.

**Figure 3:**
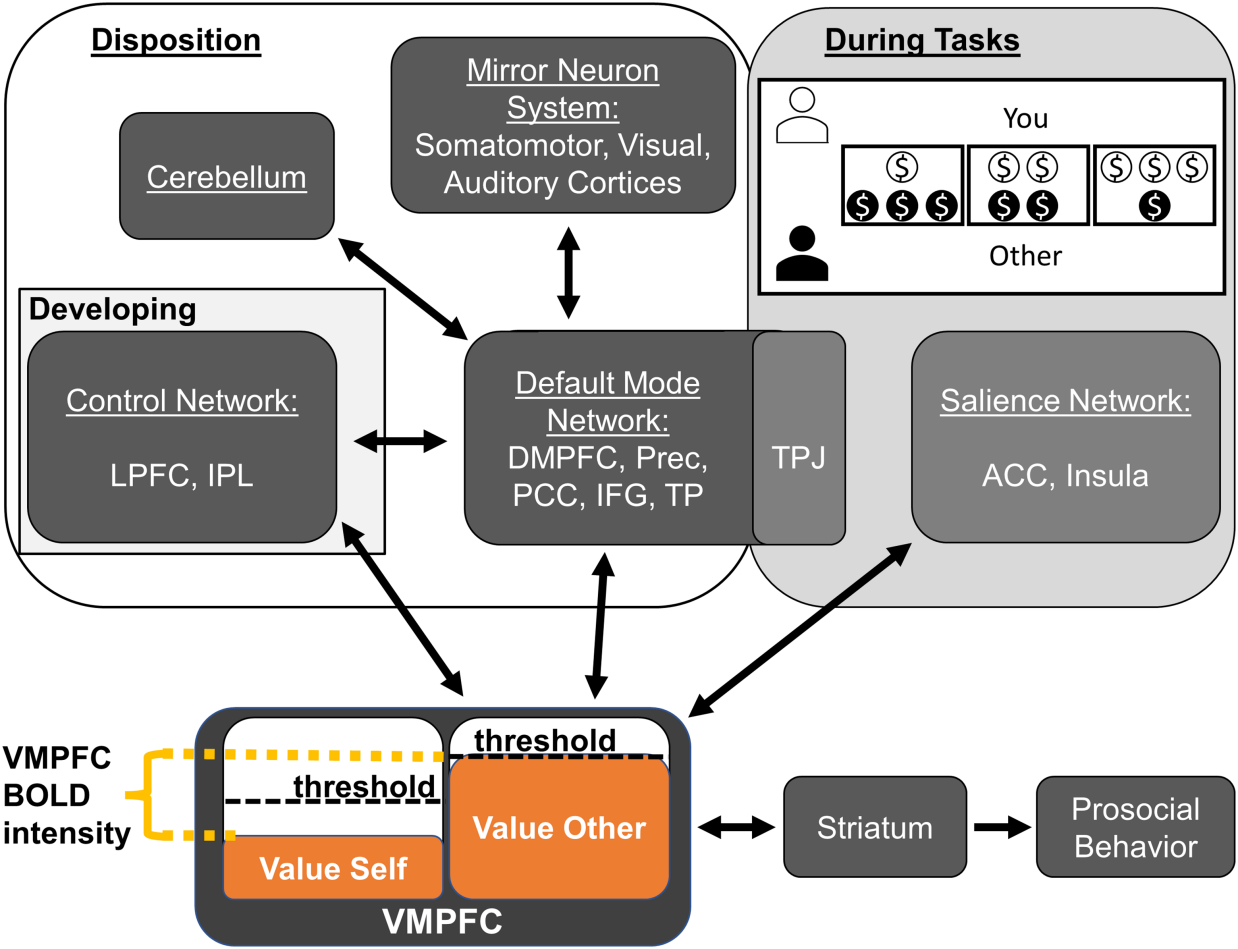
Revised Domain-General Developmental (Do-GooD) network model of prosocial cognition. We present a revised domain-general network model of prosocial cognition that expands on the previous model using the present findings in resting state brain networks. There are four key revisions. (1) Control Network and Default Mode Networks have a predicted interaction between them that develops through adolescence. (2) The Default Mode Network interfaces with the mirror neuron system in the sensory cortices. (3) The cerebellum interfaces with the Default Mode Network. (4) These aforementioned components contribute to prosocial and compassionate disposition while the TPJ and the Salience Networks are part of a module with relevance to tasks and context. As with the previous model, value accrues in the VMPFC, which has activation corresponding to the relative difference between self- and other-value. The VMPFC interfaces with the striatum to determine the threshold necessary for enacting prosocial behavior. The task shown in the “During Tasks” box is a characteristic Dictator Game. Abbreviations: anterior cingulate cortex (ACC), dorsomedial prefrontal cortex (DMPFC), inferior frontal gyrus (IFG), inferior parietal lobule (IPL), lateral prefrontal cortex (LPFC), posterior cingulate cortex (PCC), precuneus (Prec), temporal parietal junction (TPJ), temporal pole (TP), ventromedial prefrontal cortex (VMPFC).

In the following sections, we discuss these findings in the context of previous work and the Do-GooD network model of prosocial cognition; we then conclude with this study’s notable negative findings, the limitations, and suggested avenues for future work where we propose a revised Do-GooD Network Model of Prosocial Cognition (Figure 3).

### Default mode network, DLPFC, and VMPFC connectivity

The medial frontoparietal network (also known as the ‘default mode network’ or ‘DMN’) constituted the backbone of the identified subnetwork; key regions include the DMPFC, VMPFC, PCC, and precuneus, while less-defined DMN regions include the IFG-orb, and TP-sup (Uddin et al., 2019). In the identified subnetwork, the DMN within-network connectivity positively correlates with prosociality and compassion, but only between the DMPFC (a key DMN region) and the IFG-orb and TP-sup (more peripheral DMN regions). The IFG and TP are both highly relevant to social cognition (Keuken et al., 2011; Peled-Avron et al., 2019; Herlin et al., 2021), suggesting that increased within-DMN connectivity to these socially specialized regions may support DMN value-computations favoring others.

The Do-GooD network model of prosocial cognition predicts that adolescent prosociality should be supported by connections to the VMPFC from the DMN and the CN (Sipes et al., 2022). In support of this prediction, the identified subnetwork shows significant functional connectivity from the left DMPFC (a key DMN region) and the left DLPFC (a key CN region) to the VMPFC. These connections have theoretical significance to prosocial and compassionate behavior. The DMN, while having a multitude of functions, is especially relevant in social cognition (Meyer et al., 2019) and predictive coding (Dohmatob et al., 2020). The DLPFC, in contrast, represents abstract and multi-dimensional values (Dixon and Christoff, 2014), social norms (Hackel et al., 2020), and moral attitudes (Crockett et al., 2017). Connectivity from core DMN and CN regions to the left VMFPC is important because the VMPFC is known for value computation and accrual during decision-making (Boorman et al., 2009; Nicolle et al., 2012; Brosch and Sander, 2013). Taken together, one interpretation is that, during resting state activity, connections between social predictions and social rules functionally link to value-assessment in a positive relationship to adolescent prosociality and compassion.

This subnetwork also shows connectivity between DMN regions and the left DLPFC, a major region in the control network. A frontal-lobe module centers on the left superior DMPFC (DMPFC-sup), and both regions connect through the left TP-sup and the left VMPFC. The left DLPFC also connects directly to the right DMPFC, suggesting that this CN node has interconnectivity with the DMN supporting prosocial and compassionate dispositions. While this was not a direct prediction of the Do-GooD model, it suggests that these domain-general networks do not simply contribute value independently to the VMPFC but that the networks additionally have interactions related to prosociality.

Previous task-based studies on adolescent prosocially commonly highlight the VMPFC (Telzer et al., 2010; Güroglu et al., 2014; Tousignant et al., 2018; Spaans et al., 2020; Brandner et al., 2021), the DMPFC (Telzer et al., 2011; van den Bos et al., 2011; Van Hoorn et al., 2016; Will et al., 2016; Lemmers-Jansen et al., 2018; Tousignant et al., 2018; Do et al., 2019), the TP-sup (Gunther Moor et al., 2012; Tousignant et al., 2018), and the DLPFC (Telzer et al., 2011; Do et al., 2019; Spaans et al., 2020; Duell et al., 2021) as related to prosocial decision-making tasks. It is reassuring that many of the same brain regions active during prosocial tasks also form a functional subnetwork related to adolescent prosociality and compassion during rest. The alignment between task-based studies and the present subnetwork could suggest that brain activity during prosocial tasks is facilitated by an underlying network involving these frequently identified regions.

### Sensory and mirror system connectivity

The discovery of mirror neuron systems, regions that activate both for our own as well as others’ actions, was critical to understanding how the brain processes sensory information to enable social understanding through simulation (Gallese and Goldman, 1998). Sensory cortices such as somatosensory areas, the occipital lobe, the auditory cortex, as well as other areas such as the IFG, constitute mirror neuron areas (Rajmohan and Mohandas, 2007). The present findings revealed that significant interactions between DMN regions and these sensory/mirroring regions are associated with prosociality and compassion in adolescents.

Some of the most significant functional connections in the subnetwork were between the precuneus and the occipital lobe, specifically the calcarine sulci (V1) and the superior occipital gyri (OG-sup). Occipital regions connected directly to the frontal lobe, specifically, the right DMPFC connected to the left cuneus and the left DLPFC connected to the right calcarine gyrus. Major sensory regions, including the OG-sup, left auditory cortex (Heschl’s gyrus), SMA, and Rolandic operculum formed a module with the PCC, revealing a significant juncture between the core DMN and major sensing regions. These left-lateralized auditory and somatosensory regions in particular have been found to form a somatotopic auditory mirror-neuron system that was found to have increased activation related to greater empathy (Gazzola et al., 2006). Another mirror system circuit in the subnetwork links the left DMPFC-sup to the bilateral IFG-orb and the right auditory cortex. The right auditory cortex also links to the left VMPFC, which as discussed in the previous section, relates to value computation during tasks.

The Do-GooD model of prosocial cognition predicts that the DMN computes value for self and others by simulating value-predictions with sensory/mirror neuron regions. The subnetwork obtained in our study lends support to this prediction, showing that these mirroring areas interface directly with DMN nodes such as the DMPFC, PCC, and precuneus. It is worth emphasizing that, because this is resting state connectivity, these functional connections with sensory/mirror neuron areas are devoid of any explicit mirroring. This may indicate instead that adolescent mirroring during prosocial behavior is in part facilitated by at-rest engagement with internal feeling and visualization predictions, which could in turn prime mirroring to activate in moments warranting compassionate prosocial behavior. Such a mechanism would predict that adolescents who practice greater awareness of their own experiences (in such a way that increases the connectivity between the DMN and sensory/mirroring regions) could have a downstream effect of increasing their prosociality and compassion.

Sensory regions are also found in task-based contrasts studying adolescent prosocial behavior. Identified occipital lobe regions during prosocial tasks include the middle occipital gyrus (Will et al., 2018; Schreuders et al., 2019; Duell et al., 2021), the cuneus (Telzer et al., 2013; Van der Meulen et al., 2016; Lemmers-Jansen et al., 2018; Duell et al., 2021), calcarine gyrus (Telzer et al., 2010), and visual cortex generally (van den Bos et al., 2009, 2011). Somatomotor regions related to prosociality include the precentral gyrus (Okada et al., 2019; Schreuders et al., 2019; Duell et al., 2021) and SMA (Telzer et al., 2010). These sensory cortices have gained relatively less attention given their traditional interpretation as processing sense data, but a renewed interpretation as a cognitive architecture connected to the DMN for mentalizing through mirroring illuminates these regions’ significance to prosociality and compassion.

### Connectivity with the cerebellum

An unexpected yet intriguing finding this data-driven approach revealed was the dense and highly significant connectivity between the cortex and the cerebellum related to prosociality and compassion. The majority of these connections were with the bilateral precuneus, and two functional connections were with the right PCC and the left IFG-orb, respectively; as such, all cerebellar connections were to the DMN.

The cerebellum has gained mounting attention for its role in social cognition, especially with respect to mirroring, mentalizing, and abstract representations (Van Overwalle et al., 2014, 2020). Furthermore, it has been found that the posterior regions, especially Crus 1 and 2, are most involved in these mentalizing/abstraction processes (Van Overwalle et al., 2014; Guell and Schmahmann, 2020), which aligns well with their appearance in the identified subnetwork. A growing interpretation for the cerebellum’s general role is as a hub for predictive processing (Sokolov et al., 2017; Gatti et al., 2021) that generates forward internal models and action sequences (Tanaka et al., 2020).

Accordingly, given the high connectivity between the DMN and the cerebellum related to prosociality and compassion, we speculate that the cerebellum contains prosocial routines that outline prediction-oriented ‘recipes’ delivered to the DMN to be organized and deployed by other cerebral networks. It could be that during rest, readily accessing cerebellar routines prime them for use when compassionate and prosocial situations arrive in a way similar to the possible mechanism in the mirror neuron system except that it is action-oriented rather than sensation-oriented.

While previous studies find the precuneus to be significantly related to adolescent prosocial behavior (van den Bos et al., 2009, 2011; Masten et al., 2010; Güroglu et al., 2014; Van Hoorn et al., 2016; Lemmers-Jansen et al., 2018; Do et al., 2019; Spaans et al., 2020), findings relating adolescent prosociality to the cerebellum are sparse. The cerebellum has been shown to have increased activation in adolescents interacting with peers for whom they could act prosocially (Tousignant et al., 2018), making prosocial choices that involve giving other’s more freedom (Lemmers-Jansen et al., 2018), and making charitable decisions after watching a charitable peer (Duell et al., 2021). Interestingly, two of these studies also showed activation in the PCC within the same contrast (Tousignant et al., 2018; Duell et al., 2021). Unlike the majority studies that use tasks analogous to the Dictator Game, tasks showing cerebellar activation involved mentalizing about other players in a Cyberball game (Tousignant et al., 2018), thinking about a future person’s choices (Lemmers-Jansen et al., 2018), or from conforming to a prosocial peer (Duell et al., 2021), all of which suggest that the cerebellum may be involved when mentalizing connects to prosocial action.

### Negative findings

Negative findings are often under-addressed in neuroscience literature, yet they may contain meaning when discussed in the context of prior research and theory (de Graaf and Sack, 2018). Accordingly, there were five null-results we believe warrant discussion in such a context.

First, for structural connectivity, NBS failed to reject the null hypothesis that there were no significant subnetworks related to prosociality and compassion. In some ways, this also is unsurprising, since the relationship between structural connectivity and functional connectivity is complex and far from one-to-one (Honey et al., 2009; Abdelnour et al., 2014; Tsang et al., 2017). A possible reason NBS did not identify a structural subnetwork could be because structural connectivity considers spatially and metabolically constrained physical white matter pathways between regions (Bullmore and Sporns, 2012), making it less likely to demonstrate *expansive* subnetworks related to relatively subtle psychological constructs, such as prosociality and compassion, and may instead have more regional or network specific connectivity relationships not observable from this study’s data-driven approach. There is generally less evidence for structural brain changes related to prosociality in published research, with one article showing relationships to cortical thickness (Ferschmann et al., 2019) and none, to our knowledge, pertaining to structural networks.

Second, contrary to our expectation, a region that was not identified in this resting state subnetwork was the temporal parietal junction (TPJ), which has been among the most frequent regions identified in the task-based literature (for review, see Sipes et al., 2022). There are at least two important considerations that may explain why the TPJ is not included in this subnetwork. At very low test-statistic thresholds, NBS reveals that a very large number of brain regions (102 AAL regions) have functional connectivity that are at least weakly (though, significantly) related to prosocial and compassionate tendencies in adolescence, and perhaps unsurprisingly, the bilateral TPJ is included in that highly expansive, low-thresholded, network. However, we methodologically sought to highlight in this study only those regions that had connectivity with each other demonstrating a sufficiently strong relationship with prosociality, and the bilateral TPJ’s connections to this larger subnetwork did not survive thresholding. When we evaluated the thresholds at which the TPJ was lost, we found that (1) the left TPJ was pruned from the network at test-statistic = 2.25, and (2) the right TPJ was pruned earlier at test-statistic = 2.05. A possible theoretical reason for why the bilateral TPJ did not strongly connect to this prosocial subnetwork could be that it is most involved in processing contextual information within social situations (Carter and Huettel, 2013), and thus it may have a much stronger relationship to prosociality when processing in-the-moment context, such as during a task, rather than displaying enduring trait levels of prosociality as observable during resting state connectivity.

Third, the Do-GooD network model of prosocial cognition predicted connectivity from the salience network (SN) to the VMPFC, but this connection was not identified in the subnetwork (Sipes et al., 2022); however, the Do-GooD model may also offer an interpretation. A possible reason why SN regions in previous task-based activation studies (namely, the anterior cingulate cortex and the insula) were inconsistently correlated/anti-correlated with prosocial behavior could have to do with their responsibility in self-monitoring fairness in the *context* of an ongoing task, a context that differed in the tasks’ framing of the prosocial behavior (van den Bos et al., 2009, 2011; Gunther Moor et al., 2012; Do et al., 2019; Duell et al., 2021). This implies that not observing these structures in this minimal resting state subnetwork does not negate their significance, and similar to the TPJ, may indicate the SN’s alignment with a task−/situation-dependent mechanism of prosociality rather than being reflective of an adolescent’s overall prosocial and compassionate tendencies.

Fourth, we did not observe any anti-correlation between resting-state functional connectivity and prosociality and compassion. The Do-GooD model does not make any predictions about networks in which higher connectivity would be associated with lower prosociality. While some past work on adolescent prosocial neural correlates have shown negative correlations during a prosocial decision-making task towards anonymous peers (Do et al., 2019), these were in SN regions, and an absence of SN regions from both contrasts in our resting-state study may offer a tentative interpretation toward the SN’s contextual dependence.

Fifth, we did not observe statistically significant resting-state network relationships to prosociality and compassion when testing positive-correlation with only SDQ alone, and with only CEASY alone (rather than analyzing their interaction as reported in the main findings above). At a sub-threshold level, we obtained subnetworks for the SDQ and CEASY measures which, apart from measure-specific connections, both had a strong posterior, precuneus-centralized component. Interestingly, the SDQ and CEASY questionnaires share an overlapping construct ‘helping’ (Dunfield, 2014), which may further indicate potential module specificity to different constructs. Future research could benefit from testing the individual constructs separately. This may also shed light on hemispheric involvement, which may differ depending on constructs. Although it has been previously suggested that prosocial attitudes and behaviors are associated with physiological activity in the right hemisphere (Hecht, 2014), we did not observe a clear lateralization in our results with CEASY × SDQ interaction. Frontal parts of the detected subnetwork were leaning towards the left and parietal parts toward the right hemisphere (Figure 1).

### Limitations

The results of this study should be interpreted in light of its limitations.

Prosociality and compassion in this study were measured through questionnaires, not real-life behavior. While the SDQ prosocial sub-scale asks about specific behaviors, these measures are necessarily generalizations. Accordingly, it is likely that significant networks related to prosocial/compassionate actions in a relevant task would not necessarily align with the subnetwork presented here.

Another limitation involves the data-driven aspect of our analysis. While we had specific expectations inspired by the Do-GooD model of prosocial cognition in adolescents, the NBS method was agnostic to any specific *a priori* hypotheses, and thus does not represent a hypothesis driven study. However, as a first-pass toward a network analysis of resting state correlates of prosociality and compassion, we believe the data-driven approach was appropriate for this study’s exploratory aims. As mentioned above, it could have been because of this non-specific inductive approach that structural connectivity did not show any significant relationships to prosociality and compassion.

It could also be reasonable to question the meaning of a data-driven result during the resting state that highlights the DMN as a key finding when the DMN is understood as the *default mode* connectivity pattern during rest. Could it be that simply the DMN’s naturally pronounced expression during rest gives rise to spurious relationships? We believe this is unlikely for at least two reasons. First, resting state connectivity contains many network configurations that change dynamically with time (Deco et al., 2011; Yeo et al., 2011; Cole et al., 2014), and thus they are effectively averaged through taking the Pearson’s correlation across the full time series. Second, the core DMN nodes (i.e., the MPFC and the PCC) co-activate by definition as a functional network, but in the present study, these regions do *not* show functional connectivity with each other related to prosocial behavior. Conversely, many of the significant functional connections for prosociality and compassion exist between DMN nodes and non-DMN regions (i.e., LPFC, occipital lobe, auditory cortex, and cerebellum) and to non-core DMN regions (i.e., IFG-orb and TP-sup). Thus, our results reflect that adolescent prosociality and compassion may be less about core DMN activation with itself and rather its interactions with the peripheral DMN and other networks, which reduces concern of a spurious relationship to prosociality and compassion stemming from a dominant DMN during rest.

Due to the novelty of the resting state network-based approach to adolescent prosociality and compassion, our ability to contextualize the present work in the existing literature was limited to region-based findings and not network-based findings. We hope the present work may provide some initial groundwork for future studies to begin analyzing prosociality and compassion from a network perspective.

### Future work

Future work approaching prosociality and compassion should continue evaluating connectivity from both task and rest data. A useful model from previous studies is to relate fMRI data, either during rest or a task of interest, to each adolescent’s individual performance during that task, which could take place before, during, or after the MRI (Masten et al., 2010; Overgaauw et al., 2014; Tashjian et al., 2018; Tousignant et al., 2018; Spaans et al., 2020). An especially important consideration for research on adolescent prosociality is studying functional connectivity within carefully designed ecologically valid tasks, which itself constitutes a new wave of cognitive neuroscience research (Finn, 2021). Furthermore, it is worth noting that NBS has the capability to compare networks in a contrast design (Zalesky et al., 2010), allowing for possible comparisons between task-blocks within task-based fMRI data, and this design could fruitfully merge past approaches with a network-informed analysis.

To help guide these further analyses in future studies, we have revised the Do-GooD model in light of the current findings, including new specific relationships that are suggested by the present study (Figure 3). There are four key revisions: (1) CN and DMN have a predicted interaction between them; (2) The DMN interfaces with the mirror neuron system in the sensory cortices; (3) The cerebellum interfaces with the DMN; and (4) The TPJ and the Salience Networks show specific relevance to tasks and context instead of general prosocial disposition. While the present study cautiously suggests these relationships, it is important to test these model predictions specifically with *a priori* hypotheses in future studies. One explicit way to test these predictions in future resting state or task-based studies could be through Dynamic Causal Modeling (DCM), which could directly assess the validity of the Do-GooD model compared to other possible formulations (Friston et al., 2013; Sibert et al., 2022).

An important application for these findings could be to monitor this *a priori* defined functional subnetwork to assess outcomes of interventions that seek to improve prosocial behavior in adolescents. Conditions with significant deficits in prosocial behavior and compassion that may especially benefit from neuroscience guided interventions include oppositional defiant disorder (Hamilton and Armando, 2008), conduct disorder (Fairchild et al., 2019), and callous-unemotionality (Sakai et al., 2017). This work could also potentially be applied to conditions with social deficits such as autism, which also has work showing atypical networks both in fMRI (Abbott et al., 2016) and EEG (Wadhera and Kakkar, 2021), emphasizing an additional need for more multi-modal approaches in future work.

## Conclusion

The present study aimed to use the Network Based Statistic as a data-driven approach to investigate functional and structural network relationships to prosociality and compassion in adolescents. We provided novel evidence for a resting state functional connectivity subnetwork related to prosocial behavior and compassion in adolescents. Our results showed a significant subnetwork positively related to prosociality and compassion involving interactions between the DMN and the DLPFC, mirror-neuron systems, and the cerebellum. However, we did not find any such subnetworks related to structural connectivity. Overall, we found that the functional networks involved were well aligned with the Do-GooD model of prosocial cognition in adolescents and offer new testable hypotheses to further develop our understanding of prosocial development. These finding could prove useful to guide future treatment interventions seeking to improve prosocial behavior and compassion in adolescents during this important social and neurodevelopmental period.

## Data availability statement

The raw data supporting the conclusions of this article will be made available by the authors, without undue reservation.

## Ethics statement

The studies involving human participants were reviewed and approved by University of California, San Francisco Institutional Review Board (IRB). Written informed consent to participate in this study was provided by the participants' legal guardian/next of kin.

## Author contributions

BS did the conceptualization, performed the methodology, carried out the formal analysis, wrote the original draft, created the figures, and edited the manuscript. AJ and YL performed the methodology and edited the manuscript. JM edited the manuscript. TY did the conceptualization, carried out the funding acquisition, and edited the manuscript. OT did the conceptualization, carried out the funding acquisition, conducted the project administration, and edited the manuscript. All authors contributed to the article and approved the submitted version.

## Funding

This work was supported by the National Center for Complementary and Integrative Health (NCCIH) R21AT009173, R61AT009864, and R33AT009864 to OT and TY; the National Center for Advancing Translational Sciences (CTSI), National Institutes of Health, through UCSF-CTSI UL1TR001872 to OT and TY; the American Foundation for Suicide Prevention (AFSP) SRG-1-141-18 to OT and TY; UCSF Weill Institute for Neurosciences to OT and TY; UCSF Research Evaluation and Allocation Committee (REAC) and J. Jacobson Fund to OT and TY; the Fahs-Beck Fund for Research and Experimentation at The New York Community Trust to OT; the National Institute of Mental Health (NIMH) R01MH085734 and the Brain and Behavior Research Foundation (formerly NARSAD) to TY; NICHD R01HD088438 to JM.

## Conflict of interest

The authors declare that the research was conducted in the absence of any commercial or financial relationships that could be construed as a potential conflict of interest.

## Publisher’s note

All claims expressed in this article are solely those of the authors and do not necessarily represent those of their affiliated organizations, or those of the publisher, the editors and the reviewers. Any product that may be evaluated in this article, or claim that may be made by its manufacturer, is not guaranteed or endorsed by the publisher.

## Footnotes

^1^ www.fil.ion.ucl.ac.uk/spm

## Supplementary material

Supplementary Figure S1 Negative Findings. We show the results from the Network-Based Statistic (NBS) with resting state connectivity related to (A) the CEASY questionnaire alone and (B & C) the SDQ questionnaire alone. All these results were non-significant at the test-statistic threshold 2.75. Network number of edges, nodes, and NBS-corrected p-values are displayed in the bottom right panel of each network. Note that both the networks in (A) and (C) contain a highly overlapping precuneus centered module, with the right precuneus highlighted in red.

## References

[ref1] Abbott A. E. Nair A. Keown C. L. Datko M. Jahedi A. Fishman I.. (2016). Patterns of atypical functional connectivity and behavioral links in autism differ between default, salience, and executive networks. Cereb. Cortex 26, 4034–4045. doi: 10.1093/cercor/bhv191, PMID: 26351318 PMC5027998

[ref2] Abdelnour F. Voss H. U. Raj A. (2014). Network diffusion accurately models the relationship between structural and functional brain connectivity networks. NeuroImage 90, 335–347. doi: 10.1016/j.neuroimage.2013.12.039, PMID: 24384152 PMC3951650

[ref3] Boorman E. D. Behrens T. E. Woolrich M. W. Rushworth M. F. (2009). How green is the grass on the other side? Frontopolar cortex and the evidence in favor of alternative courses of action. Neuron 62, 733–743. doi: 10.1016/j.neuron.2009.05.014, PMID: 19524531

[ref4] Brandner P. Güro˘glu B. van de Groep S. Spaans J. P. Crone E. A. (2021). Happy for us not them: differences in neural activation in a vicarious reward task between family and strangers during adolescent development. Dev. Cogn. Neurosci. 51:100985. doi: 10.1016/j.dcn.2021.100985, PMID: 34273748 PMC8319462

[ref5] Brosch T. Sander D. (2013). Neurocognitive mechanisms underlying value-based decision-making: from core values to economic value. Front. Hum. Neurosci. 7:398. doi: 10.3389/fnhum.2013.00398, PMID: 23898252 PMC3721023

[ref7] Bullmore E. Sporns O. (2012). The economy of brain network organization. Nat. Rev. Neurosci. 13, 336–349. doi: 10.1038/nrn3214 22498897

[ref8] Carter R. M. Huettel S. A. (2013). A nexus model of the temporal-parietal junction. Trends Cogn. Sci. 17, 328–336. doi: 10.1016/j.tics.2013.05.007, PMID: 23790322 PMC3750983

[ref9] Cole M. W. Bassett D. S. Power J. D. Braver T. S. Petersen S. E. (2014). Intrinsic and task-evoked network architectures of the human brain. Neuron 83, 238–251. doi: 10.1016/j.neuron.2014.05.014, PMID: 24991964 PMC4082806

[ref10] Crockett M. J. Siegel J. Z. Kurth-Nelson Z. Dayan P. Dolan R. J. (2017). Moral transgressions corrupt neural representations of value. Nat. Neurosci. 20, 879–885. doi: 10.1038/nn.4557, PMID: 28459442 PMC5462090

[ref11] de Graaf T. A. Sack A. T. (2018). When and how to interpret null results in NIBS: A taxonomy based on prior expectations and experimental design. Front. Neurosci. 12:915. doi: 10.3389/fnins.2018.00915, PMID: 30618550 PMC6297282

[ref12] Deco G. Jirsa V. K. McIntosh A. R. (2011). Emerging concepts for the dynamical organization of resting-state activity in the brain. Nat. Rev. Neurosci. 12, 43–56. doi: 10.1038/nrn2961, PMID: 21170073

[ref13] Dixon M. L. Christoff K. (2014). The lateral prefrontal cortex and complex value-based learning and decision making. Neurosci. Biobehav. Rev. 45, 9–18. doi: 10.1016/j.neubiorev.2014.04.011, PMID: 24792234

[ref14] Do K. T. McCormick E. M. Telzer E. H. (2019). The neural development of prosocial behavior from childhood to adolescence. Soc. Cogn. Affect. Neurosci. 14, 129–139. doi: 10.1093/scan/nsy117, PMID: 30608610 PMC6382927

[ref15] Dohmatob E. Dumas G. Bzdok D. (2020). Dark control: The default mode network as a reinforcement learning agent. Hum. Brain Mapp.. 41, 3318–3341 32500968 10.1002/hbm.25019 PMC7375062

[ref16] Duell N. van Hoorn J. McCormick E. M. Prinstein M. J. Telzer E. H. (2021). Hormonal and neural correlates of prosocial conformity in adolescents. Dev. Cogn. Neurosci. 48:100936. doi: 10.1016/j.dcn.2021.100936, PMID: 33611148 PMC7903062

[ref17] Dunfield K. A. (2014). A construct divided: prosocial behavior as helping, sharing, and comforting subtypes. Front. Psychol. 5:958. doi: 10.3389/fpsyg.2014.00958, PMID: 25228893 PMC4151454

[ref18] Eisenberg N. Zhou Q. Koller S. (2001). Brazilian adolescents’ prosocial moral judgment and behavior: relations to sympathy, perspective taking, gender-role orientation, and demographic characteristics. Child Dev. 72, 518–534. doi: 10.1111/1467-8624.00294 11333082

[ref21] Fairchild G. Hawes D. J. Frick P. J. Copeland W. E. Odgers C. L. Franke B.. (2019). Conduct disorder. Nat. Rev. Dis. Primers. 5, 1–25. doi: 10.1038/s41572-019-0095-y 31249310

[ref22] Ferschmann L. Vijayakumar N. Grydeland H. Overbye K. Sederevicius D. Due-Tønnessen P.. (2019). Prosocial behavior relates to the rate and timing of cortical thinning from adolescence to young adulthood. Dev. Cogn. Neurosci. 40:100734. doi: 10.1016/j.dcn.2019.100734, PMID: 31739096 PMC6974908

[ref23] Finn E. S. (2021). Is it time to put rest to rest? Trends Cogn. Sci. 25, 1021–1032. doi: 10.1016/j.tics.2021.09.005, PMID: 34625348 PMC8585722

[ref24] Friston K. Moran R. Seth A. K. (2013). Analysing connectivity with granger causality and dynamic causal modelling. Curr. Opin. Neurobiol. 23, 172–178. doi: 10.1016/j.conb.2012.11.010, PMID: 23265964 PMC3925802

[ref25] Gallese V. Goldman A. (1998). Mirror neurons and the simulation theory of mind-reading. Trends Cogn. Sci. 2, 493–501. doi: 10.1016/S1364-6613(98)01262-5, PMID: 21227300

[ref26] Gatti D. Rinaldi L. Ferreri L. Vecchi T. (2021). The human cerebellum as a hub of the predictive brain. Brain Sci. 11:1492. doi: 10.3390/brainsci11111492, PMID: 34827491 PMC8615481

[ref27] Gazzola V. Aziz-Zadeh L. Keysers C. (2006). Empathy and the Somatotopic auditory Mirror system in humans. Curr. Biol. 16, 1824–1829. doi: 10.1016/j.cub.2006.07.072, PMID: 16979560

[ref28] Gilbert P. Catarino F. Duarte C. Matos M. Kolts R. Stubbs J.. (2017). The development of compassionate engagement and action scales for self and others. J. Compassionate Health Care 4:4. doi: 10.1186/s40639-017-0033-3

[ref29] Goodman R. (2001). Psychometric properties of the strengths and difficulties questionnaire. J. Am. Acad. Child Adolesc. Psychiatry 40, 1337–1345. doi: 10.1097/00004583-200111000-00015 11699809

[ref30] Guell X. Schmahmann J. (2020). Cerebellar functional anatomy: A didactic summary based on human fMRI evidence. Cerebellum 19, 1–5. doi: 10.1007/s12311-019-01083-9, PMID: 31707620

[ref31] Gunther Moor B. Güroglu B. Op de Macks Z. A. Rombouts S. A. Van der Molen M. W. Crone E. A. (2012). Social exclusion and punishment of excluders: neural correlates and developmental trajectories. NeuroImage 59, 708–717. doi: 10.1016/j.neuroimage.2011.07.028, PMID: 21791248

[ref32] Güroglu B. Will G.-J. Crone E. A. (2014). Neural correlates of advantageous and disadvantageous inequity in sharing decisions (H. Rao, Ed.). PLoS One 9:e107996. doi: 10.1371/journal.pone.0107996, PMID: 25238541 PMC4169616

[ref33] Hackel L. M. Wills J. A. Van Bavel J. J. (2020). Shifting prosocial intuitions: neurocognitive evidence for a value-based account of group-based cooperation. Soc. Cogn. Affect. Neurosci. 15, 371–381. doi: 10.1093/scan/nsaa055, PMID: 32337604 PMC7308656

[ref34] Hamilton S. Armando J. (2008). Oppositional defiant disorder. Am. Fam. Physician 78, 861–866. 18841736

[ref35] Hecht D. (2014). Cerebral lateralization of pro- and anti-social tendencies. Exp. Neurobiol. 23, 1–27. doi: 10.5607/en.2014.23.1.1, PMID: 24737936 PMC3984952

[ref36] Herlin B. Navarro V. Dupont S. (2021). The temporal pole: from anatomy to function—A literature appraisal. J. Chem. Neuroanat. 113:101925. doi: 10.1016/j.jchemneu.2021.101925, PMID: 33582250

[ref37] Honey C. J. Sporns O. Cammoun L. Gigandet X. Thiran J. P. Meuli R.. (2009). Predicting human resting-state functional connectivity from structural connectivity. Proc. Natl. Acad. Sci. 106, 2035–2040. doi: 10.1073/pnas.0811168106, PMID: 19188601 PMC2634800

[ref39] Inagaki T. K. Eisenberger N. I. (2016). Giving support to others reduces sympathetic nervous system- related responses to stress. Psychophysiology 53, 427–435. doi: 10.1111/psyp.12578, PMID: 26575283

[ref40] Keuken M. C. Hardie A. Dorn B. T. Dev S. Paulus M. P. Jonas K. J.. (2011). The role of the left inferior frontal gyrus in social perception: an rTMS study. Brain Res. 1383, 196–205. doi: 10.1016/j.brainres.2011.01.073, PMID: 21281612

[ref41] Lemmers-Jansen I. L. Krabbendam L. Amodio D. M. Van Doesum N. J. Veltman D. J. Van Lange P. A. (2018). Giving others the option of choice: an fMRI study on low-cost cooperation. Neuropsychologia 109, 1–9. doi: 10.1016/j.neuropsychologia.2017.12.009, PMID: 29221833

[ref42] Masten C. L. Eisenberger N. I. Pfeifer J. H. Dapretto M. (2010). Witnessing peer rejection during early adolescence: neural correlates of empathy for experiences of social exclusion. Soc. Neurosci. 5, 496–507. doi: 10.1080/17470919.2010.490673, PMID: 20602283 PMC2957502

[ref43] Meyer M. L. Davachi L. Ochsner K. N. Lieberman M. D. (2019). Evidence that default network connectivity during rest consolidates social information. Cereb. Cortex 29, 1910–1920. doi: 10.1093/cercor/bhy071, PMID: 29668862

[ref44] Moieni M. Irwin M. R. Haltom K. E. B. Jevtic I. Meyer M. L. Breen E. C.. (2019). Exploring the role of gratitude and support-giving on inflammatory outcomes. Emotion 19, 939–949. doi: 10.1037/emo0000472, PMID: 30265078

[ref45] Nicolle A. Klein-Flügge M. C. Hunt L. T. Vlaev I. Dolan R. J. Behrens T. E. (2012). An agent independent Axis for executed and modeled choice in medial prefrontal cortex. Neuron 75, 1114–1121. doi: 10.1016/j.neuron.2012.07.023, PMID: 22998878 PMC3458212

[ref46] Okada N. Yahata N. Koshiyama D. Morita K. Sawada K. Kanata S.. (2019). Neurometabolic and functional connectivity basis of prosocial behavior in early adolescence. Sci. Rep. 9:732. doi: 10.1038/s41598-018-38355-z, PMID: 30679738 PMC6345858

[ref47] Overgaauw S. Güroglu B. Rieffe C. Crone E. A. (2014). Behavior and neural correlates of empathy in adolescents. Dev. Neurosci. 36, 210–219. doi: 10.1159/000363318 24993549

[ref48] Peled-Avron L. Glasner L. Gvirts H. Z. Shamay-Tsoory S. G. (2019). The role of the inferior frontal gyrus in vicarious social touch: A transcranial direct current stimulation (tDCS) study. Dev. Cogn. Neurosci. 35, 115–121. doi: 10.1016/j.dcn.2018.04.010, PMID: 29773509 PMC6968961

[ref49] Rajmohan V. Mohandas E. (2007). Mirror neuron system. Indian J. Psychiatry 49, 66–69. doi: 10.4103/0019-5545.31522, PMID: 20640069 PMC2900004

[ref50] Rubinov M. Sporns O. (2010). Complex network measures of brain connectivity: uses and interpretations. NeuroImage 52, 1059–1069. doi: 10.1016/j.neuroimage.2009.10.003, PMID: 19819337

[ref51] Sakai J. T. Dalwani M. S. Mikulich-Gilbertson S. K. Raymond K. McWilliams S. Tanabe J.. (2017). Imaging decision about whether to benefit self by harming others: adolescents with conduct and substance problems, with or without callous-unemotionality, or developing typically. Psychiatry Res. Neuroimaging 263, 103–112. doi: 10.1016/j.pscychresns.2017.03.004, PMID: 28371655 PMC5705947

[ref52] Schreuders E. Klapwijk E. T. Will G.-J. Güroglu B. (2018). Friend versus foe: neural correlates of prosocial decisions for liked and disliked peers. Cogn. Affect. Behav. Neurosci. 18, 127–142. doi: 10.3758/s13415-017-0557-1, PMID: 29318509 PMC5823968

[ref53] Schreuders E. Smeekens S. Cillessen A. H. Gürog˘lu B. (2019). Friends and foes: neural correlates of prosocial decisions with peers in adolescence. Neuropsychologia 129, 153–163. doi: 10.1016/j.neuropsychologia.2019.03.004, PMID: 30871971

[ref54] Sibert C. Hake H. S. Stocco A. (2022). The structured mind at rest: low-frequency oscillations reflect interactive dynamics between spontaneous brain activity and a common architecture for task control. Front. Neurosci. 16:832503. doi: 10.3389/fnins.2022.832503, PMID: 35898414 PMC9309720

[ref55] Sipes B. S. Yang T. T. Parks K. C. Jariwala N. Tymofiyeva O. (2022). A domain-general develop-mental “Do-GooD” network model of prosocial cognition in adolescence: a systematic review. Front. Behav. Neurosci. 16:815811. doi: 10.3389/fnbeh.2022.815811, PMID: 35350389 PMC8957975

[ref57] Sokolov A. A. Miall R. C. Ivry R. B. (2017). The cerebellum: adaptive prediction for movement and cognition. Trends Cogn. Sci. 21, 313–332. doi: 10.1016/j.tics.2017.02.005, PMID: 28385461 PMC5477675

[ref58] Spaans J. P. Peters S. Crone E. A. (2020). Neural reward related-reactions to monetar gains for self and charity are associated with donating behavior in adolescence. Soc. Cogn. Affect. Neurosci. 15, 151–163. doi: 10.1093/scan/nsaa027, PMID: 32163162 PMC7304510

[ref59] Tanaka H. Ishikawa T. Lee J. Kakei S. (2020). The Cerebro-cerebellum as a locus of forward model: A review. Front. Syst. Neurosci. 14:19. doi: 10.3389/fnsys.2020.00019, PMID: 32327978 PMC7160920

[ref60] Tashjian S. M. Weissman D. G. Guyer A. E. Galván A. (2018). Neural response to prosocial scenes relates to subsequent giving behavior in adolescents: a pilot study. Cogn. Affect. Behav. Neurosci. 18, 342–352. doi: 10.3758/s13415-018-0573-9, PMID: 29464552 PMC8320692

[ref61] Telzer E. H. Fuligni A. J. Lieberman M. D. Galván A. (2013). Ventral striatum activation to prosocial re-wards predicts longitudinal declines in adolescent risk taking. Dev. Cogn. Neurosci. 3, 45–52. doi: 10.1016/j.dcn.2012.08.004, PMID: 23245219 PMC4598077

[ref62] Telzer E. H. Masten C. L. Berkman E. T. Lieberman M. D. Fuligni A. J. (2010). Gaining while giving: an fMRI study of the rewards of family assistance among white and Latino youth. Soc. Neurosci. 5, 508–518. doi: 10.1080/17470911003687913, PMID: 20401808 PMC3079017

[ref63] Telzer E. H. Masten C. L. Berkman E. T. Lieberman M. D. Fuligni A. J. (2011). Neural regions associated with self control and mentalizing are recruited during prosocial behaviors towards the family. NeuroImage 58, 242–249. doi: 10.1016/j.neuroimage.2011.06.013, PMID: 21703352 PMC3276247

[ref64] Tousignant B. Eugène F. Sirois K. Jackson P. L. (2018). Difference in neural response to social exclusion observation and subsequent altruism between adolescents and adults. Neuropsychologia 116, 15–25. doi: 10.1016/j.neuropsychologia.2017.04.017, PMID: 28412511

[ref65] Tsang A. Lebel C. A. Bray S. L. Goodyear B. G. Hafeez M. Sotero R. C.. (2017). White matter structural connectivity is not correlated to cortical resting-state functional connectivity over the healthy adult lifespan. Front. Aging Neurosci. 9:144. doi: 10.3389/fnagi.2017.00144, PMID: 28572765 PMC5435815

[ref67] Tzourio-Mazoyer N. Landeau B. Papathanassiou D. Crivello F. Etard O. Delcroix N.. (2002). Automated anatomical labeling of activations in SPM using a macroscopic anatomical Parcellation of the MNI MRI single-subject brain. NeuroImage 15, 273–289. doi: 10.1006/nimg.2001.0978, PMID: 11771995

[ref68] Uddin L. Q. Yeo B. T. T. Spreng R. N. (2019). Towards a universal taxonomy of macro-scale functional human brain networks. Brain Topogr. 32, 926–942. doi: 10.1007/s10548-019-00744-6, PMID: 31707621 PMC7325607

[ref69] van den Bos W. van Dijk E. Westenberg M. Rombouts S. A. Crone E. A. (2009). What motivates repayment? Neural correlates of reciprocity in the trust game. Soc. Cogn. Affect. Neurosci. 4, 294–304. doi: 10.1093/scan/nsp009, PMID: 19304843 PMC2728629

[ref70] van den Bos W. van Dijk E. Westenberg M. Rombouts S. A. Crone E. A. (2011). Changing brains, changing perspectives: the neurocognitive development of reciprocity. Psychol. Sci. 22, 60–70. doi: 10.1177/0956797610391102, PMID: 21164174

[ref71] Van der Meulen M. van Ijzendoorn M. H. Crone E. A. (2016). Neural correlates of prosocial behavior: compensating social exclusion in a four-player Cyberball game. PLoS One 11:e0159045. doi: 10.1371/journal.pone.0159045, PMID: 27391364 PMC4938540

[ref72] Van Hoorn J. Van Dijk E. Güro˘glu B. Crone E. A. (2016). Neural correlates of prosocial peer influence on public goods game donations during adolescence. Soc. Cogn. Affect. Neurosci. 11, 923–933. doi: 10.1093/scan/nsw013, PMID: 26865424 PMC4884312

[ref73] Van Overwalle F. Baetens K. Mariën P. Vandekerckhove M. (2014). Social cognition and the cerebel-lum: a meta-analysis of over 350 fMRI studies. NeuroImage 86, 554–572. doi: 10.1016/j.neuroimage.2013.09.033, PMID: 24076206

[ref74] Van Overwalle F. Manto M. Cattaneo Z. Clausi S. Ferrari C. Gabrieli J. D. E.. (2020). Consensus paper: cerebellum and social cognition. Cerebellum 19, 833–868. doi: 10.1007/s12311-020-01155-1, PMID: 32632709 PMC7588399

[ref76] Wadhera T. Kakkar D. (2021). Social cognition and functional brain network in autism spectrum disorder: insights from EEG graph-theoretic measures. Biomed. Signal Process. Control 67:102556. doi: 10.1016/j.bspc.2021.102556

[ref77] Wang R. Benner T. Sorensen A. G. Wedeen V. J. (2007). Diffusion toolkit: a software package for diffusion imaging data processing and tractography. Proc. Intl. Soc. Mag. Reson. Med. 15:3720.

[ref78] Whitfield-Gabrieli S. Nieto-Castanon A. (2012). Conn: A functional connectivity toolbox for correlated and Anticorrelated brain networks. Brain Connect. 2, 125–141. doi: 10.1089/brain.2012.0073, PMID: 22642651

[ref79] Will G.-J. Crone E. A. Lier P. A. C. V. Güroglu B. (2018). Longitudinal links between childhood peer acceptance and the neural correlates of sharing. Dev. Sci. 21:e12489. doi: 10.1111/desc.12489, PMID: 27753220 PMC5763347

[ref80] Will G.-J. Crone E. A. van Lier P. A. Güroglu B. (2016). Neural correlates of retaliatory and prosocial reactions to social exclusion: associations with chronic peer rejection. Dev. Cogn. Neurosci. 19, 288–297. doi: 10.1016/j.dcn.2016.05.004, PMID: 27261927 PMC6988598

[ref1001] Xia M. Wang J. He Y. (2013). BrainNet Viewer: a network visualization tool for human brain connectomics. PloS one 8:e68910. doi: 10.1371/journal.pone.0068910, PMID: 23861951 PMC3701683

[ref81] Yeo B. T. Krienen F. M. Sepulcre J. Sabuncu M. R. Lashkari D. Hollinshead M.. (2011). The organization of the human cerebral cortex estimated by intrinsic functional connectivity. J. Neurophysiol. 106, 1125–1165. doi: 10.1152/jn.00338.2011, PMID: 21653723 PMC3174820

[ref82] Yuan J. P. Henje Blom E. Flynn T. Chen Y. Ho T. C. Connolly C. G.. (2019). Test–retest reliability of graph theoretic metrics in adolescent brains. Brain Connect. 9, 144–154. doi: 10.1089/brain.2018.0580, PMID: 30398373 PMC6444894

[ref83] Zalesky A. Fornito A. Bullmore E. T. (2010). Network-based statistic: identifying differences in brain networks. NeuroImage 53, 1197–1207. doi: 10.1016/j.neuroimage.2010.06.041, PMID: 20600983

[ref84] Zhong J. Rifkin-Graboi A. Ta A. T. Yap K. L. Chuang K.-H. Meaney M. J.. (2014). Functional networks in parallel with cortical development associate with executive functions in children. Cereb. Cortex 24, 1937–1947. doi: 10.1093/cercor/bht051, PMID: 23448875

