# Supplementary figures and images for: Resting state brain subnetwork relates to prosociality and compassion in adolescents
Source: Front Psychol. 2022 Oct 20;13:1012745. doi: 10.3389/fpsyg.2022.1012745 (PMC9632179; doi:10.3389/fpsyg.2022.1012745)

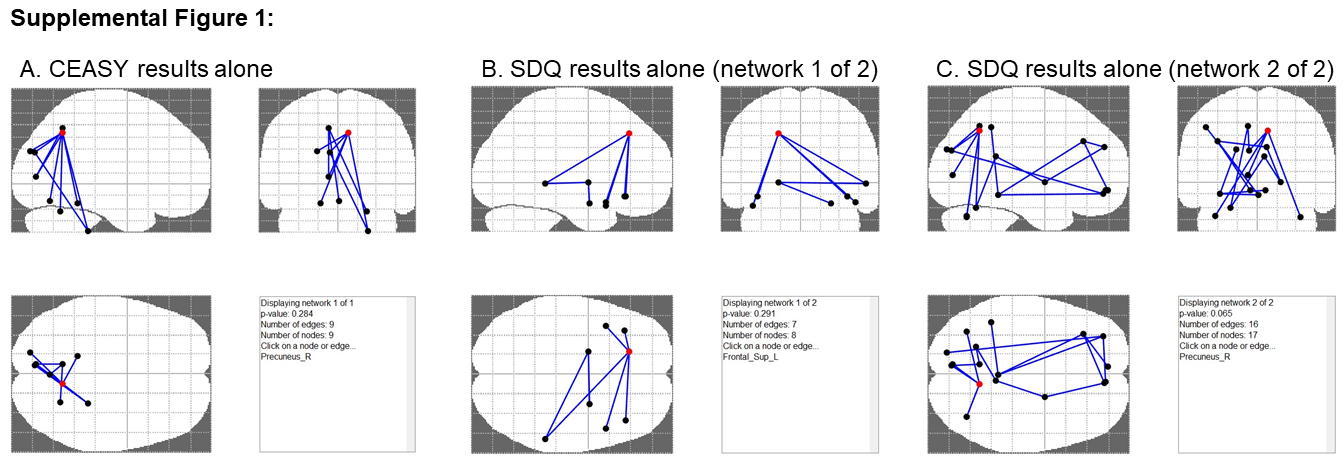

Supplement: Supplementary Figure S1 — Negative Findings. We show the results from the Network-Based Statistic (NBS) with resting state connectivity related to (A) the CEASY questionnaire alone and (B & C) the SDQ questionnaire alone. All these results were non-significant at the test-statistic threshold 2.75. Network number of edges, nodes, and NBS-corrected p-values are displayed in the bottom right panel of each network. Note that both the networks in (A) and (C) contain a highly overlapping precuneus centered module, with the right precuneus highlighted in red. [file Image_1.tif]
